# Kenny Music Performance Anxiety Inventory: Contribution for the Portuguese Validation

**DOI:** 10.3390/bs12020018

**Published:** 2022-01-23

**Authors:** Pedro Dias, Lurdes Veríssimo, Nânci Figueiredo, Patrícia Oliveira-Silva, Sofia Serra, Daniela Coimbra

**Affiliations:** 1 Faculty of Education and Psychology, Universidade Católica Portuguesa, 4169-005 Porto, Portugal; lverissimo@ucp.pt (L.V.); nanci.figueiredo.psi@hotmail.com (N.F.); posilva@ucp.pt (P.O.-S.); 2 CEDH-Research Centre for Human Development, 4169-005 Porto, Portugal; 3 CITAR-Research Centre for Science and Technology of the Arts, School of Arts, Universidade Católica Portuguesa, 4169-005 Porto, Portugal; sserra@ucp.pt; 4 i2ADS—Research Institute of Art, Design and Society, School of Music and Performing Arts, Polytechnic Institute of Porto, 4000-045 Porto, Portugal; DanielaCoimbra@esmae.ipp.pt

**Keywords:** music performance anxiety, assessment, K-MPAI, validation, psychometric properties

## Abstract

(1) Background: The aim of the present study was to contribute to the validation of the Portuguese version of the Kenny Music Performance Anxiety Inventory (K-MPAI) and to study its psychometric properties. (2) Methods: A sample of 164 undergraduate music students in Portugal (62.2% female; mean age = 22.63; SD = 4.36) completed an online survey composed of the K-MPAI Portuguese version, the State Trait Anxiety Inventory, and a sociodemographic questionnaire. The K-MPAI psychometric properties were examined using exploratory factor analyses, known-group differences, and Cronbach’s alpha. (3) Results: A four-factor structure was identified, in line with recent validation of this measure in other countries: music performance anxiety-related symptoms, depression and hopelessness, parental support, and memory self-efficacy. Concurrent and known-group validity were established, and reliability scores were appropriate for the dimensions and total score. (4) Conclusions: The results provide initial evidence of the appropriateness of the Portuguese version of the K-MPAI.

## 1. Introduction

Music performance anxiety (MPA) is defined as the experience of feeling anxious and apprehensive about one’s music performance skills in a severe and persistent way in a music performance context when this distress is not justified by the individual’s ability and level of preparation. It is frequently associated with a setting where there is a high investment, an evaluation situation, and a consequent possibility of failure [1]. Although many other professions may also be associated with high anxiety levels, some evidence suggests that musicians display more symptoms of performance anxiety than the general working population [2].

MPA is one of the most frequently described disorders among musicians [3,4], with recent literature reporting prevalence ranging between 24% and 70% of orchestra musicians [5]. MAP can affect musicians in all stages of professional trajectory with different levels of experience, practice, and musical level of attainment [6]. There are different degrees of severity, and musicians suffering from MPA often display emotional (e.g., anxious apprehension towards a performance), cognitive (e.g., focused attention on fear), somatic (e.g., increased heart rate or shaking hands), and behavioural symptoms (e.g., avoiding auditions, solos, and rehearsals) [1].

With regard to its aetiology, Barlow’s model [7] suggests that MPA could arise by the presence or interaction of three types of vulnerabilities that influence the degree of the anxiety response: (i) generalised biological vulnerability, explained by biological factors that influence the development of negative emotions; (ii): generalised psychological vulnerability, based on early experiences that induce a perception that certain events are uncontrollable; and (iii) specific psychological vulnerability, when the experience of feeling anxious occurs due to specific environmental stimuli which are reinforced by different types of learning [5,6,7,8,9].

Research in MPA has focused on several predisposing individual, social, and situational factors, such as age, sex, motivation, personality traits, audience presence, type of instrument, performance setting, repertoire, and level of demand [1,10,11,12,13,14,15]. The results of some of these studies indicate a predisposition of women to feel higher levels of dysfunctional anxiety in the contexts of musical performance [16,17]. Extrinsic motivation (e.g., meeting parental expectations) [18] and personality characteristics (e.g., higher levels of trait anxiety or high perfectionism) [19] are also predictors of performance anxiety. Studies also showed the central role of social and situational variables on MPA, suggesting significantly higher anxiety levels when the performance has an audience, highlighting concerns such as fear of being negatively judged, the size and status of the audience, and the competitive nature of the performance [11,12,18].

While some musicians manifest adaptive and focused anxiety, many others experience deep and prolonged physical and psychological suffering, which impacts the quality of performance [1]. Thus, a valid and reliable tool for assessing MPA is crucial to identify musicians in need of intervention to manage their anxiety effectively and to study this phenomenon in different cultures [1]. 

One of the most-used instruments developed to assess MPA is the Kenny Music Performance Anxiety Inventory (K-MPAI) [8]. Based on Barlow’s model [7] adapted to MPA, K-MPAI assesses symptoms of anxiety, memory bias, negative cognitions related to MPA, and elements related to personal history (e.g., primary experiences during development) (Kenny, 2011). The first version of the K-MPAI includes 26 items [8]. A revised version contains 40 items [20].

The psychometric properties of the revised version of the K-MPAI [20] were analysed with sample populations of professional and amateur musicians and music university students. This version was adapted and validated in several countries (e.g., Spain, Brazil, Germany, Australia, Peru, and Romania) [6,20,21,22,23]. Through these validation studies, different factorial structures were tested. Recent studies have tested the factor structure of K-MPAI using 30 of the original 40 items in Peru and Australia, and in Romania [6,24]. In the Romanian version, a four-factor structure showed appropriate psychometric properties [6].

Assessing MPA in adult musicians and music students is essential for screening and intervention design purposes. Considering this need and the absence of a valid measure for such assessment in Portugal, the present study aimed to contribute to the validation of the K-MPAI in Portugal and to perform the first study of its psychometric properties in a sample of university music students.

## 2. Materials and Methods

### 2.1. Procedures

For the K-MPAI translation and validation procedures to Portuguese, the criteria proposed by the International Test Commission [25] were followed: a bilingual expert performed a process of translation and a different expert performed a blind back-translation from the original K-MPAI version to achieve linguistic equivalence; then, the research team reached an agreement about the best version of the instrument in terms of comprehension, conceptualisation, content, semantics, and culture. Moreover, a think-aloud focus group involving 14 music master’s students and instrumentalists changed 12 items to meet their suggestions regarding clarity and comprehension for the target sample (K-MPAI Form Available online: https://www.researchgate.net/publication/299461895_Kenny Music Performance Anxiety Inventory K-MPAI and scoring form (accessed on 01 November 2021)).

Participants were recruited through an online invitation sent through the coordinators of degree programs in music higher education institutions from all the regions of Portugal. Participants who agreed to participate responded to an online survey implemented via the survey platform Qualtrics^®^.Available online: https://www.qualtrics.com/ (accessed on 14 September 2020).

Complete ethical assessments and approvals were sought in advance of the project. All subjects gave their informed consent for inclusion before they participated in the study. They were informed that participation in the study was voluntary, that all the information gathered would be confidential and anonymous, and about their right to withdrawal at any time. Only the research team had access to the database, stored safely in a university-owned computer with password protection. The study was conducted in accordance with the Declaration of Helsinki, and the protocol was approved by the Scientific Board of the Faculty of Education and Psychology in September 2019.

Inclusion criteria were age greater than 18 years old and less than 40 years old, and enrolment in an instrument degree at a higher education institution in Portugal. These criteria were defined in order to obtain a diverse sample regarding age, country regions, and instruments played. A total of 336 responses to the online survey were obtained. However, 172 responses were excluded due to incomplete protocols and the presence of participants who did not fulfil the inclusion criteria.

### 2.2. Participants

The study included data on 164 undergraduate music students (62.2% female) from diverse higher education institutions from different regions in Portugal (north, centre, and south). Students’ age ranged from 18 to 39 years old (M = 22.63; SD = 4.36). The characterisation of participants’ music-related variables is shown in Table 1.

Participants were asked about receiving previous professional psychological support due to anxiety. In all, 51 (31.1%) reported having had anxiety-related support in the past, while 111 (67.7%) said they did not. When addressing drug intake, the data showed that 50 participants reported taking or having taken the following anxiety-related medications: anxiolytics (*n* = 19), antidepressants (*n* = 6), beta-blockers (*n* = 17), other drugs (e.g., valerian; cannabis tea; *n* = 15).

### 2.3. Instruments

#### 2.3.1. Kenny Music Performance Anxiety Inventory (K-MPAI) 

K-MPAI [6] is a 40-item instrument that assesses MPA based on Barlow’s [7] triple vulnerability model that accounts for the development of anxiety or mood disorders in general [8], as discussed previously. Each item can be rated on a 7-point Likert scale, ranging from 0 (strongly disagree) to 6 (strongly agree). A total score can be obtained by summing up all the items, with higher scores indicating higher anxiety and psychological distress levels (e.g., item 4: “I often find it difficult to work up the energy to do things”; item 10: “Prior to, or during a performance, I get feelings akin to panic”). 

#### 2.3.2. State Trait Anxiety Inventory (STAI); Portuguese Version 

This self-report questionnaire is one of the most widely used instruments to measure anxiety in adults [26,27]. STAI has two independent scales, one to assess state anxiety (STAI-S) and another to evaluate trait anxiety (STAI-T), each with 20 items. The STAI-S is composed of items that capture psychological and physiological transient or situational anxiety (e.g., item 17: “I am tense; I am worried”), while STAI-T is composed of items that capture individual differences associated with a tendency to experience anxiety, which are relatively stable over time (e.g., item 13: “I wish I could be as happy as others seem to be”). Each item can be rated on a 4-point Likert scale, ranging from 1 (almost never) to 4 (almost always). A total score can be obtained by summing up all the items, with higher scores indicating higher anxiety and psychological distress levels [27]. The STAI Portuguese version [26,27] showed high internal consistency, with a Cronbach alpha of 0.88 for both scales [27].

#### 2.3.3. Sociodemographic Questionnaire

The sociodemographic questionnaire collected data such as (i) sex; (ii) age; (iii) year of degree (e.g., first, second, or third undergraduate year); (iv) instrument played; (v) participation in ensemble activities, rehearsal frequency (e.g., weekly, biweekly, or occasionally), and function (e.g., conductor, section leader, instrumentalist); (vi) participation in music competitions (e.g., in the past year, national or international); (vii) instrument practice time per week; and (viii) history of psychological support and medicine intake due to anxiety (e.g., in anticipation or immediately before the performance, regularity). 

### 2.4. Data Analysis Overview

The data were imported from the Qualtrics platform to the Statistical Package for Social Sciences [SPSS], version 26.0 [28]. The sociodemographic data (sex, age, year of degree, and instrument played) were analysed using descriptive statistics such as mean, frequency, and percentage.

According to the objectives of the present study, the K-MPAI psychometric properties were examined for validity and reliability. An exploratory factor analysis (EFA) using principal component analysis (PCA) with the varimax rotation method was carried out to determine the factor structure of the data, based on the Romanian validation results for the K-MPAI [6], in which the authors considered 30 out of the 40 items of the original instrument. The suitability of the sample’s data to perform the EFA was evaluated using Keizer–Meyer–Olkin tests (KMO; a measure of sampling adequacy) and Bartlett’s test of sphericity (general significance of all correlations) [29]. Pearson’s correlation coefficient was used to assess concurrent validity by comparing the K-MPAI results with the STAI (state and trait) results. 

To analyse differences between groups with normally distributed data, the independent samples *t*-test was used for (i) sex differences, (ii) participants with vs. without a history of professional support due to anxiety, and (iii) participants with vs. without medicine intake to manage anxiety-related symptoms. These analyses allowed us to test known-group validity, as differences in MPA are expected to occur between male and female participants, with female students scoring higher than males, and with participants with previous support for anxiety reasons (professional support and medicine intake) also scoring higher than participants without a history of such support.

Cronbach’s alpha coefficient was used to assess the internal consistency.

## 3. Results

### 3.1. Validity—Factorial Structure of the K-MPAI

The KMO value was 0.845, suggesting the adequacy of the sample for factor analysis (Field, 2005). Bartlett’s test of sphericity reported a significant value, χ2 (435) = 2247.436, *p* < 0.001, confirming that the correlation matrix was appropriate (Field, 2005).

A four-factor solution was a suitable option in terms of the explained variance and the items’ factor loading. The final structure proposed for the instrument is composed of the following factors: Factor 1—MPA-related symptoms (e.g., item 15: “Thinking about the evaluation I may get interferes with my performance”); Factor 2—depression and hopelessness (e.g., item 4: “I often find it difficult to work up the energy to do things”); Factor 3—parental support (e.g., item 9: “My parents were mostly responsive to my needs”); and Factor 4—memory self-efficacy (e.g., item 37: “I am confident playing from memory”). The four-factor model can be seen in Table 2.

The four factors together accounted for 50.63% of the variance. Factor 1 explained 23.17% of the variance and comprised 18 items (10, 11, 12, 14, 15, 16, 18, 20, 21, 22, 24, 25, 26, 28, 30, 34, 36, and 38). Factor 2 explained 12.52% of the variance and comprised seven items (3, 4, 6, 13, 19, 27, and 31). Factor 3 explained 7.80% of the variance and comprised three items (9, 23, and 33). Finally, Factor 4 explained 7.15% of the variance and comprised two items (36 and 37).

### 3.2. Internal Consistency 

The Portuguese version of K-MPAI, with a Cronbach’s alpha coefficient of 0.91, showed high overall internal consistency for the 30 total items. For all the factors individually, this coefficient was higher than 0.75, with a Cronbach’s α = 0.99 for Factor 1, α = 0.79 for Factor 2, α = 0.76 for Factor 3, and α = 0.89 for Factor 4. These results suggest that the proposed instrument is reliable for this sample [30].

### 3.3. Concurrent Validity: Correlation of K-MPAI Scores with STAI Scores

To determine the concurrent validity, a Pearson correlation analysis was also performed between K-MPAI and STAI data. The results indicate a significant positive correlation between the scores of the two measures, showing that participants who present higher levels of anxiety in STAI (particularly in the STAI-T) also present greater MPA according to K-MPAI scores. The Pearson correlation analysis is shown in Table 3.

### 3.4. Music Anxiety Performance—Group Differences

Regarding the analysis of gender differences, a *t*-test revealed a statistically significant difference between males and females in relation to the degree of MPA. Female participants showed more significant symptoms related to MPA (Factor 1) and higher levels of MPA in general (total score) compared to male participants, *t* (164) = −3.40, *p* < 0.001, and *t* (160) = −2.83, *p* < 0.01, respectively (see Table 4).

Differences between participants with and without a history of psychological support due to anxiety problems were also calculated, showing a statistically significant difference. Participants who reported having had anxiety-related professional support showed higher levels of symptoms related to MPA (Factor 1), greater symptoms of depression and hopelessness (Factor 2), and a higher global level of MPA (total score) compared to participants who reported never having had professional help, *t* (162) = 3.28, *p* < 0.01, *t* (161) = 3.86, *p* < 0.001, and *t* (158) = 3.50, *p* < 0.001, respectively (see Table 5).

Finally, a *t*-test was applied to compare participants with and without medicine intake to manage anxiety-related symptoms, revealing a statistically significant difference. Participants who reported taking or having taken medication because of anxiety symptoms showed higher levels of MPA-related symptoms (Factor 1), higher levels of depression and hopelessness (Factor 2), less parental support (Factor 3), and greater MPA in general compared to those who reported never taking medication for anxiety, *t* (164) = 3.84, *p* < 0.001, *t* (163) = 4.28, *p* < 0.001, *t* (136) = −1.98, *p* < 0.05, and *t* (160) = 4.57, *p* < 0.001, respectively (see Table 6).

## 4. Discussion

The central goal of this study was to contribute to the validation of K-MPAI for the Portuguese adult population.

Following recent adaptations of the K-MPAI [6], an exploratory factor analysis was conducted, considering 30 items and the extraction of four factors. The results showed that a four-factor structure in the Portuguese population was adequate. This structure—MPA-related symptoms (F1), depression and hopelessness (F2), parental support (F3), and memory self-efficacy (F4)—considering the large percentage of the variance explained and item loadings on each factor, ensure the construct validity of this version. In addition, the Portuguese version of K-MPAI demonstrated high levels of reliability in the four factors and total score, in line with the values obtained in the study by Faur et al. [27], with the same factorial structure.

These results support the redefinition of the factorial structure of the K-MPAI in terms of the number of items and factors, in accordance with publications suggesting the use of 30 of the original 40 items [6,24].

As shown in previous studies [8,31], trait and state anxiety were positively associated with MPA, supporting the concurrent validity of this version of K-MPAI: participants who evidenced higher levels of trait and state anxiety showed higher levels of MPA.

Female participants showed higher levels of MPA-related symptoms (F1) and global levels of MPA when compared with male participants. These results are consistent with the literature, indicating that women tend to report more anxiety than men [32,33,34]. The study of the differences between participants with and without a history of professional follow-up due to anxiety problems showed that the participants who reported a professional intervention for anxiety problems showed higher levels of MPA-related symptoms (F1), depression and hopelessness (F2), and global levels of MPA. Regarding the differences found between participants with and without a history of medication use for anxiety, the participants who reported using medication for anxiety presented higher levels of MPA-related symptoms (Factor 1), depression and hopelessness (F2), and global levels of MPA and less parental support (Factor 3) compared to those who reported never taking medication for anxiety. These results suggest that a history of previous anxiety problems is associated with higher levels of MPA, in line with research indicating that trait anxiety is a risk factor for the development of MPA [35]. Taken together, known-group differences reinforce the construct validity of the Portuguese version of the K-MPAI.

## 5. Conclusions

This was the first study of the psychometric properties of the K-MPAI in the Portuguese population. The results concerning validity and reliability were appropriate and consistent with recent validation studies of this instrument in other countries. The study of the concurrent validity and known-group differences contribute to a deeper understanding of MPA.

Additional research with the Portuguese version of the K-MPAI is still needed, considering that this is a relevant tool for researchers and psychologists working with musicians and music students, allowing an appropriate screening of anxiety related to musical performance. Future studies must include larger samples, enabling the use of confirmatory factor analysis (CFA) in the Portuguese version of the K-MPAI, as well as the study of different populations (e.g., professional musicians). It is also relevant to further examine the specificity of two of the K-MPAI dimensions (depression and hopelessness and memory self-efficacy) and their relationship with demographic variables. Finally, taking into consideration the latest developments on this instrument and this study’s results, future studies combining data from different cultures could provide additional evidence supporting the appropriateness of a revised and shorter version of the K-MPAI.

The existence of a robust and validated instrument that assesses music performance anxiety is a powerful contribution to music teaching and learning. The Portuguese version of the K-MPAI will allow, in the context of higher education, the assessment and monitoring of students’ anxiety, and, consequently, the appropriate management of its impact on performance quality. This is particularly important in this stage of professional training, considering that students must develop their music skills, but also skills to cope with stressful performance situations.

## Figures and Tables

**Table 1:** Characterisation of participants.

		*n* = 164	%
Undergraduate year			
	1st year	29	17.7%
	2nd year	39	23.8%
	3rd year	81	49.4%
	Not reported	15	9.1%
Instruments played			
	Woodwind	50	30.5%
	Brass	41	25%
	String	35	21.3%
	Keyboard	19	11.6%
	Voice	15	9.1%
	Percussion	3	1.8%
	Not reported	1	0.7%
Another instrument			
	Yes	45	27.3%
	No	118	72%
	Not reported	1	0.7%
Involvement in ensemble activities (group/band/orchestra)			
	Yes	127	77.3%
	No	35	21.3%
	Not reported	2	1.4%
Participation in music competitions			
	Yes	129	78.7%
	No	35	21.3%
	National	84	51.2%
	International	38	23.2%
Weekly instrument practice time			
	<11 h	46	28%
	11–20 h	50	35.4%
	>20 h	47	28.7%
	Not reported	21	7.9%

**Table 2:** Results of exploratory factor analysis for the K-MPAI and factor loadings of the 30 items (final version).

K-MPAI Items	Factor 1	Factor 2	Factor 3	Factor 4
	MPA-Related Symptoms	Depression and Hopelessness	Parental Support	Memory Self-Efficacy
38. I am concerned about being scrutinised by others.	**0.757**			
18. I am often concerned about a negative reaction from the audience.	**0.739**			
26. My worry and nervousness about my performance interferes with my focus and concentration.	**0.701**			
15. Thinking about the evaluation I may get interferes with my performance.	**0.687**			
10. Prior to, or during a performance, I get feelings akin to panic.	**0.656**	0.296		
30. Prior to, or during a performance, I have increased muscle tension.	**0.648**			
28. I often prepare for a concert with a sense of dread and impending disaster.	**0.618**	0.462		
34. I worry so much before a performance, I cannot sleep.	**0.617**	0.206		
11. I never know before a concert whether I will perform well.	**0.612**	0.243		
22. Prior to, or during a performance, I experience increased heart rate like pounding in my chest.	**0.607**			
14. During a performance, I find myself thinking about whether I’ll get through it.	**0.604**	0.371		−0.211
21. I worry that one bad performance may ruin my career.	**0.592**			
16. Prior to, or during a performance, I feel sick or faint or have a churning in my stomach.	**0.587**	0.349		
36. Prior to, or during a performance, I experience shaking or trembling or tremor.	**0.573**	0.272		
24. I give up worthwhile performance opportunities due to anxiety.	**0.525**			
25. After the performance, I worry about whether I played well enough.	**0.453**			−0.264
20. From early in my music studies, I remember being anxious about performing.	**0.452**			
3. Sometimes I feel depressed without knowing why.		**0.716**		
13. I often feel that I am not worth much as a person.	0.259	**0.700**		
4. I often find it difficult to work up the energy to do things.		**0.693**		−0.266
6. I often feel that life has not much to offer me.		**0.681**		
31. I often feel that I have nothing to look forward to.	0.261	**0.590**		
19. Sometimes I feel anxious for no particular reason.	0.338	**0.483**		
12. Prior to, or during a performance, I experience dry mouth.	**0.257**	0.289		−0.275
23. My parents always listened to me.		−0.223	**0.862**	
9. My parents were mostly responsive to my needs.			**0.811**	
33. My parents encouraged me to try new things.			**0.687**	
27. As a child, I often felt sad.		**0.469**	−0.489	
35. When performing without music, my memory is reliable.				**0.915**
37. I am confident playing from memory.				**0.872**
R^2^ (%)	23.17%	12.52%	7.80%	7.15%

Notes: bold characters indicate items retained in each factor.

**Table 3:** Pearson correlation between STAI and K-MPAI factors.

	MPA-Related Symptoms	Depression and Hopelessness	Parental Support	Memory Self-Efficacy	K-MPAI Total Score
STAI_Y1	0.40 ***	0.64 ***	−0.32 ***	−0.07	0.52 ***
STAI_Y2	0.53 ***	0.78 ***	−0.24 **	−0.11	0.67 ***

** *p* < 0.01; *** *p* < 0.001.

**Table 4:** Gender differences related to MPA (dimensions and total score).

	Gender		
	Male	Female	
	(n)	(n)	
	Mean (SD)	Mean (SD)	
Factor 1 MPA-related symptoms	62 3.11 (1.23)	102 3.74 (1.11)	*t* (164) = −3.40 ***
Factor 2 Depression and hopelessness	61 2.70 (1.29)	102 2.81 (1.20)	*t* (163) = −0.54
Factor 3 Parental support	62 4.22 (1.33)	101 4.01 (1.39)	*t* (163) = 0.95
Factor 4 Memory self-efficacy	62 3.27 (1.96)	102 2.92 (1.91)	*t* (164) = 1.15
Total Score (K-MPAI)	61 2.86 (1.01)	101 3.30 (0.94)	*t* (160) = −2.83 **

** *p* < 0.01; *** *p* < 0.001.

**Table 5:** Differences between participants with and without a history of anxiety-related professional support (K-MPAI dimensions and total score).

	Anxiety-Related Professional Support		
	With	Without	
	(n)	(n)	
	Mean (SD)	Mean (SD)	
Factor 1 MPA-related symptoms	51 3.96 (1.14)	111 3.31 (1.17)	t (162) = 3.28 **
Factor 2 Depression and hopelessness	51 3.31 (1.30)	110 2.53 (1.12)	t (161) = 3.86 ***
Factor 3 Parental support	51 4.07 (1.34)	110 4.09 (1.39)	t (161) = −0.11
Factor 4 Memory self-efficacy	51 3.18 (1.88)	111 2.98 (1.97)	t (162) = 0.61
Total Score(K-MPAI)	51 3.53 (0.93)	109 2.96 (0.97)	t (158) = 3.50 ***

** *p* < 0.01; *** *p* < 0.001.

**Table 6:** Differences between participants with and without a history of medicine intake to manage anxiety symptoms (dimensions and total score).

	Anxiety Medicine Intake		
	Yes	No	
	(n)	(n)	
	Mean (SD)	Mean (SD)	
Factor 1 MPA-related symptoms	50 4.03 (0.99)	114 3.28 (1.21)	t (164) = 3.84 ***
Factor 2 Depression and hopelessness	50 3.36 (1.13)	113 2.51 (1.19)	t (163) = 4.28 ***
Factor 3 Parental support	50 3.77 (1.39)	113 4.23 (1.35)	t (163) = −1.98 *
Factor 4 Memory self-efficacy	50 2.84 (2.06)	114 3.14 (1.88)	t (164) = −0.93
Total Score (K-MPAI)	50 3.63 (0.89)	112 2.91 (0.95)	t (160) = 4.57 ***

* *p* < 0.05; *** *p* < 0.001.

## Data Availability

The data presented in this study are available on request from the corresponding author.
